# Berberine Improves Glucose and Lipid Metabolism in Obese Mice Through the Reduction of IRE1/GSK-3β Axis-Mediated Inflammation

**DOI:** 10.2174/0118715303319434241113161606

**Published:** 2025-01-08

**Authors:** Lina Ding, Jingjing Xia, Hua Wang, Junyi Qian, Xiaodan Jin, Yang Yang, Jing Xia, Wenbin Shang, Ming Chen

**Affiliations:** 1 Department of Endocrinology, Jiangyin Hospital Affiliated to Nanjing University of Chinese Medicine, No. 130 Renmin Middle Road, Jiangyin City, Jiangsu Province, 214413, China;; 2 Department of Otolaryngology, Jiangyin Hospital Affiliated to Nanjing University of Chinese Medicine, No. 130 Renmin Middle Road, Jiangyin City, Jiangsu Province, 214413, China;; 3 First School of Clinical Medicine, Nanjing University of Chinese Medicine, No. 138 Xianlin Avenue, Nanjing City, Jiangsu Province, 210023, China

**Keywords:** Berberine, glucose and lipid metabolism, IRE1/GSK-3β axis, inflammation, insulin resistance, obesity

## Abstract

**Introduction:**

Berberine (BBR) has the characteristics of repressing hyperglycemia, obesity, and inflammation, as well as improving insulin resistance. However, the underlying mechanism remains to be fully understood. This study explores whether BBR regulates inositol requiring enzyme 1 (IRE1)/glycogen synthase kinase 3 beta (GSK-3β) axis to resist obesity-associated inflammation, thereby improving glucolipid metabolism disorders.

**Methods:**

Mice were fed a high-fat diet and administrated with BBR, followed by measurement of weight change, biochemical indicators, as well as glucose and insulin tolerance. Insulin-resistant 3T3-L1 adipocyte models were established, and the model cells were treated with BBR and IRE1 inhibitors. Cell viability was detected by cell counting kit-8 assay. Inflammatory factor secretion and glucose consumption were measured *via* specific kits. Oil red O staining was used to observe lipid droplet formation, and protein expressions in the IRE1/GSK-3β axis were determined *via* Western blot.

**Results:**

BBR reduced weight, insulin resistance, levels of triglyceride, total cholesterol, free fatty acid, high-density lipoprotein, and low-density lipoprotein but improved glucose tolerance in obese mice. BBR and IRE1 inhibitors demonstrated no cytotoxicity. BBR and IRE1 inhibitors diminished secretion of tumor necrosis factor-alpha, interleukin-6, and monocyte chemoattractant protein 1, lipid droplet formation, and values of p-IRE1/IRE1 and p-GSK-3β/GSK-3β, but elevated glucose consumption in insulin-resistant adipocytes.

**Conclusion:**

BBR improves glucose and lipid metabolism in obese mice through the reduction of IRE1/GSK-3β axis-mediated inflammation, showing the great potential of BBR in reversing insulin resistance in obesity.

## 1 INTRODUCTION

Currently, obesity is becoming a global health issue rather than only a problem in developed countries [1]. Long-term chronic obesity causes many metabolic-related disorders, including nonalcoholic fatty liver disease, type 2 diabetes, cardiovascular diseases, some types of cancer, retinopathy, osteoarthritis, and, in extreme cases, myocardial infarction and stroke [2].

In obese patients with excess visceral adipose tissues, adipocyte hypertrophy and proliferation trigger local hypoxia and oxidative stress, which, in turn, leads to the massive secretion of cytokines and adipokines by the adipocytes, promoting the aggregation of immune cells, thus resulting in the inflammatory response [3, 4]. Under the promotion of cytokines, tumor necrosis factor-alpha (TNF-α), interleukin-1 (IL-1), IL-6, IL-8, and monocyte chemoattractant protein1 (MCP1), various immune cells are accumulated in obese adipose tissues, including proinflammatory macrophages and B cells, while regulatory T cells, natural killer T cells, and eosinophils are declined [5]. Those aggregated immune cells will release more cytokines, and superfluous fatty acids and cytokines disturb intracellular insulin signaling, leading to insulin resistance [6, 7]. As a result, the vicious cycle between insulin resistance and metabolic inflammation forms and induces lipid and carbohydrate metabolism disorders [8, 9].

In recent years, several studies have suggested that endoplasmic reticulum stress (ERS) is involved in the inflammation of adipocytes in the occurrence and development of obesity [10-12]. Under the stimulation of ERS in adipocytes, the over-activated ER unfolded protein response (UPR) interferes with normal adipose tissue metabolism, intensifies adipose tissue inflammatory response, promotes adipokine secretion, and affects adipose tissue browning and thermogenesis pathways [13]. Inositol requiring enzyme 1 (IRE1), as one of the three pathways in UPR induction, has been reported to link ER dysfunction to obesity-associated inflammation [14]. Following the knockout of IRE1, mice with a high- fat diet (HFD) develop metabolic disturbances characterized by hepatic steatosis and insulin resistance. In 3T3-L1 adipocytes, inhibition of IRE1 kinase activity prevents the activation of nuclear factor-kappa-B (NF-κB), the secretion of IL-6, and adipocyte catabolism [15]. In addition, in the ERS state, IRE1α has been found to induce the activation of glycogen synthase kinase 3 beta (GSK-3β), a multifunctional Ser/Thr kinase, which promotes the transcription and secretion of inflammatory cytokines IL-1β and TNF-α, suggesting that the activation of GSK-3β may be involved in the activation of the NF-κB-mediated inflammatory pathway [16].

Some natural compounds have displayed promising effects on glucolipid metabolism and diabetes mellitus. Berberine (BBR) is a natural isoquinoline alkaloid extracted from *Coptis chinensis* in traditional Chinese medicine, with the characteristics of anti-hyperglycemia, anti-obesity, anti-inflammation, and insulin resistance promotion [17]. Mechanically, BBR inhibits ERS *via* GSK-3β [18, 19] and is thought to improve diabetes mellitus and glucolipid metabolism disorders induced by liver injury by blocking ERS by GSK-3β [20, 21]. However, the underlying mechanism remains to be fully understood. Hence, this study aims to explore whether BBR regulates the IRE1/GSK-3β axis to resist obesity-associated inflammation, thereby improving glucolipid metabolism disorders.

## 2 MATERIALS AND METHODS

### 2.1 Animals and Ethics Statement

Eight-week-old male C57BL/6J mice (24-26 g, n=18) were housed in a specific pathogen-free laboratory at 22-24°C with 50-60% humidity and a 12 h light/dark cycle, with 2 or 3 mice in a cage. All mice had free access to food and water. Involved animal experiments were conducted based on the guidelines of the China Council on Animal Care and Use Health, and this study was approved by the Ethics Committee of Jiangyin Hospital of Traditional Chinese Medicine for Experimental Animals Welfare (No. 202303).

### 2.2 Construction of Obesity Models and Pharmacological Treatment

After a week of environmental adaptation, C57BL/6J mice were randomly assigned into the Sham group, HFD group, and HFD/BBR group (n = 6/group). Mice in the latter two groups were fed with a 45 kcal% fat diet (D12451, Research Diets, New Brunswick, New Jersey, USA) for 12 weeks [22] and injected intragastrically with sterile saline solution (S8776, Sigma-Aldrich, St. Louis, Missouri, USA) or BBR (50 mg/kg; HY-N0716, MedChemExpress, Shanghai, China) dissolved in sterile saline solution, respectively, twice a week [23]. Mice in the Sham group received a normal diet (D11112201, Research Diets, USA) without treatment. During the BRR administration, the weight of all mice was monitored every two weeks during the 8-week period.

### 2.3 Glucose and Insulin Tolerance Tests

Mice were fasted for 6 h, and the mice on the fat diet were subjected to an injection of 0.8 g/kg glucose or 0.75 U/kg insulin, and mice with normal diet were given an injection of 1.2 g/kg glucose or 0.75 U/kg insulin [24]. Blood was collected by tail incision after injection at 0, 20, 40, 60, 80, 100, and 120 min, and then glucose level was detected on an Accu-Chek Aviva glucometer (Hoffman-La Roche, Basel, Switzerland).

### 2.4 Biochemical Measurement

At the end of the 8-week treatment, mice underwent 12 h fasting, and blood was collected from the inferior vena cava of mice and centrifuged to obtain the serum. Sigma-Aldrich (USA) offered kits for triglyceride (TG, MAK266), total cholesterol (TC, MAK043), free fatty acid (FFA, MAK044), high-density lipoprotein (HDL, MAK045), and low-density lipoprotein (LDL, MAK045), and the kit for glycosylated hemoglobin (GHb, BC5615) was purchased from Solarbio (China). As per the instructions, standards and serum samples were added into corresponding wells of microplates, followed by 30-60 min of incubation with prepared Master Reaction Mix away from light at 37°C or room temperature (for TG determination). The absorbance of each microplate was measured at 570 nm in Multiskan™ FC Microplate Photometer (51119180ET, Thermo Scientific, Waltham, Massachusetts, USA). Finally, mice were anesthetized with 150 mg/kg of sodium pentobarbital (P3761, Haoran Biological Technology, Shanghai, China; intraperitoneal injection) and sacrificed by dislocation of the cervical vertebra.

### 2.5 Reagents

Dexamethasone (Dex, ID0170), 3-isobutyl-1-methylxanthine (IBMX, II0010), and STF-083010 (YS154978), a specific IRE1α I endonuclease inhibitor, were purchased from Solarbio (Beijing, China) and collocated into stock solution with dimethyl sulfoxide (DMSO, D8371, Solarbio, China) before use. BBR was also dissolved into DMSO for use.

### 2.6 Cell Culture and Differentiation Induction

Mouse embryonic fibroblast cell line, 3T3-L1 (AW- CELLS-M0036, AnWei-sci, Shanghai, China), was cultured in Dulbecco's modified eagle medium (DMEM, BasMed-AW-010, AnWei-sci, China) containing 10% fetal bovine serum (FBS, AW-FBS-001, AnWei-sci, China) and 1% Penicillin-Streptomycin (V900929, Sigma-Aldrich, USA). All 3T3-L1 cells were incubated in 96-well plates (3×10^4^ cells/cm^2^) and placed in an incubator with a temperature of 37°C and an air condition of 5% CO_2_. Two days after cell confluence, media were replaced with complete DMEM as described above and then supplemented with 10 µg/mL insulin (I3536, Sigma-Aldrich, USA), 0.25 µM Dex, and 0.5 mM IBMX to induce differentiation of 3T3-L1 cells at 37°C for two days. Afterward, differentiated 3T3-L1 cells were cultured in complete DMEM containing 10 µg/mL insulin for two days and then placed into complete DMEM without insulin for 4 days, with media changed every two days [25].

### 2.7 Modeling of Insulin-Resistant Adipocytes and Cell Grouping

Differentiated 3T3-L1 cells were divided into different groups. 1) Blank group: normal culture; 2) Model group: 1 µM Dex stimulation for 48 h; 3) Model+STF-08301 group: 6-h treatment of 100 µM STF-08301 in growth DMEM, followed by 1 µM Dex stimulation for 48 h; and 4) Model+BBR group: 48-h treatment of 5 μM BBR after 1 µM Dex stimulation for 48 h. In detail, 3T3-L1 cells (3×10^4^ cells/cm^2^) were seeded in 96-well plates and incubated with 1 µM Dex in complete DMEM at 37°C for 48 h to construct insulin-resistant adipocyte models [25]. STF-08301 was diluted with growth DMEM into a concentration of 100 µM to treat differentiated 3T3-L1 cells for 6 h before modeling [26]. BBR was added into growth DMEM to treat insulin-resistant model adipocytes for 48 h at a final concentration of 5 μM [27].

### 2.8 Cell Counting Kit-8 (CCK-8) Assay

Cell viability was determined using a CCK-8 kit (ab228554, Abcam, Cambridge, UK). In detail, adipocytes were cultured in 96-well plates at a density of 5×10^3^ cells/well, followed by incubation with WST-8 Solution (10 µL/well) away from light at 37°C for 4 h. Finally, Multiskan™ FC Microplate Photometer was used to measure the absorbance of cells in each well at 460 nm.

### 2.9 Inflammatory Cytokine and Glucose Assay

Mouse enzyme-linked immunosorbent assay (ELISA) kits for TNF-α (PT513), IL-6 (PI326), and MCP1 (PC125) were purchased from Beyotime (Shanghai, China). According to the instructions, 50 µL/well culture supernatant of adipocytes and standards were added into corresponding wells of microplates supplemented with 50 µL/well diluted buffer, followed by a 2 h incubation at room temperature. Afterward, a biotinylated antibody (100 µL/well) was added to incubate cells for 1 h. Next, 100 µL/well horseradish peroxidase (HRP)-labeled streptavidin was kept in wells for 15 min without light, and TMB buffer (100 µL/well) was kept in the reaction system for 10 min in the dark. After the stop solution (50 µL/well) was added, the absorbance at 450 nm was detected in the Multiskan™ FC Microplate Photometer immediately.

The level of glucose in the culture supernatant of adipocytes was determined *via* a glucose assay kit (glucose oxidase method, A154-1-1, Nanjing Jiancheng Bioengineering Institute, Nanjing, China). In brief, samples and standards were incubated with a determination working solution in 96-well microplates at 37°C for 10 min, and then Multiskan™ FC Microplate Photometer was used to detect the absorbance at 505 nm of each well.

### 2.10 Oil Red O Staining

After adipocytes were treated as described above, the culture medium was removed, and cells were washed with phosphate-buffered saline (PBS, abs962, Absin, Shanghai, China). Subsequently, adipocytes were fixed in 10% paraformaldehyde (120449, Prosperich, Guangzhou, China) at room temperature for 40 min. Following PBS washing, 50 µL/well Oil red O (O8010, Solarbio, China) was applied to stain adipocytes for 30 min. Adipocytes were washed with pure water, followed by observation of lipid droplet generation under the light of ×200 magnification *via* a fluorescence confocal microscope (IX71, OLYMPUS, Tokyo, Japan).

### 2.11 Western Blot

RIPA Buffer (R0010, Solarbio, China) supplemented with a mixture of protease and phosphatase inhibitors (P1261, Solarbio, China) was employed to extract proteins from adipocytes. Following a 5 minute boiling water bath, protein concentration was determined by the BCA Protein Assay Kit (PC0020, Solarbio, China). With the assistance of sodium dodecyl sulfate-polyacrylamide gel electrophoresis (SDS-PAGE), proteins were separated in NuPAGE™ 4-12%, Bis-Tris (NP0321BOX, Thermo Scientific, USA). Polyvinylidene fluoride (PVDF) membranes (YA1701, Solarbio, China) were loaded with the separated proteins and blocked with Blocking Buffer (37581, Thermo Scientific, USA) at room temperature for 1 h. Then, the membranes were sequentially incubated with primary antibodies at 4°C overnight and secondary antibodies at room temperature for 1 h. Following the treatment of BeyoECL Plus (P0018S, Beyotime, China), the band signals were analyzed using a 5200 imaging system (Tanon, Shanghai, China). Data analysis was carried out with the help of Image J software (1.52s version, National Institutes of Health, Bethesda, Maryland, USA), with GAPDH as the internal reference.

The information of involved antibodies from Abcam (UK) is listed below: primary antibodies against p-IRE1 (ab48187, 118 kDa, 1:1000), IRE1 (ab37073,117 kDa, 1:1000), p-GSK-3β (ab45383, 50 kDa, 1:1000), GSK-3β (ab93926, 44 kDa, 1:1000), and GAPDH (ab8245, 32 kDa, 1:1000); secondary antibodies: HRP-coupled goat anti-rabbit IgG (ab205718, 1:2000) and goat anti-mouse IgG (ab205719, 1:2000).

### 2.12 Statistical Analyses

**Fig. (1):**
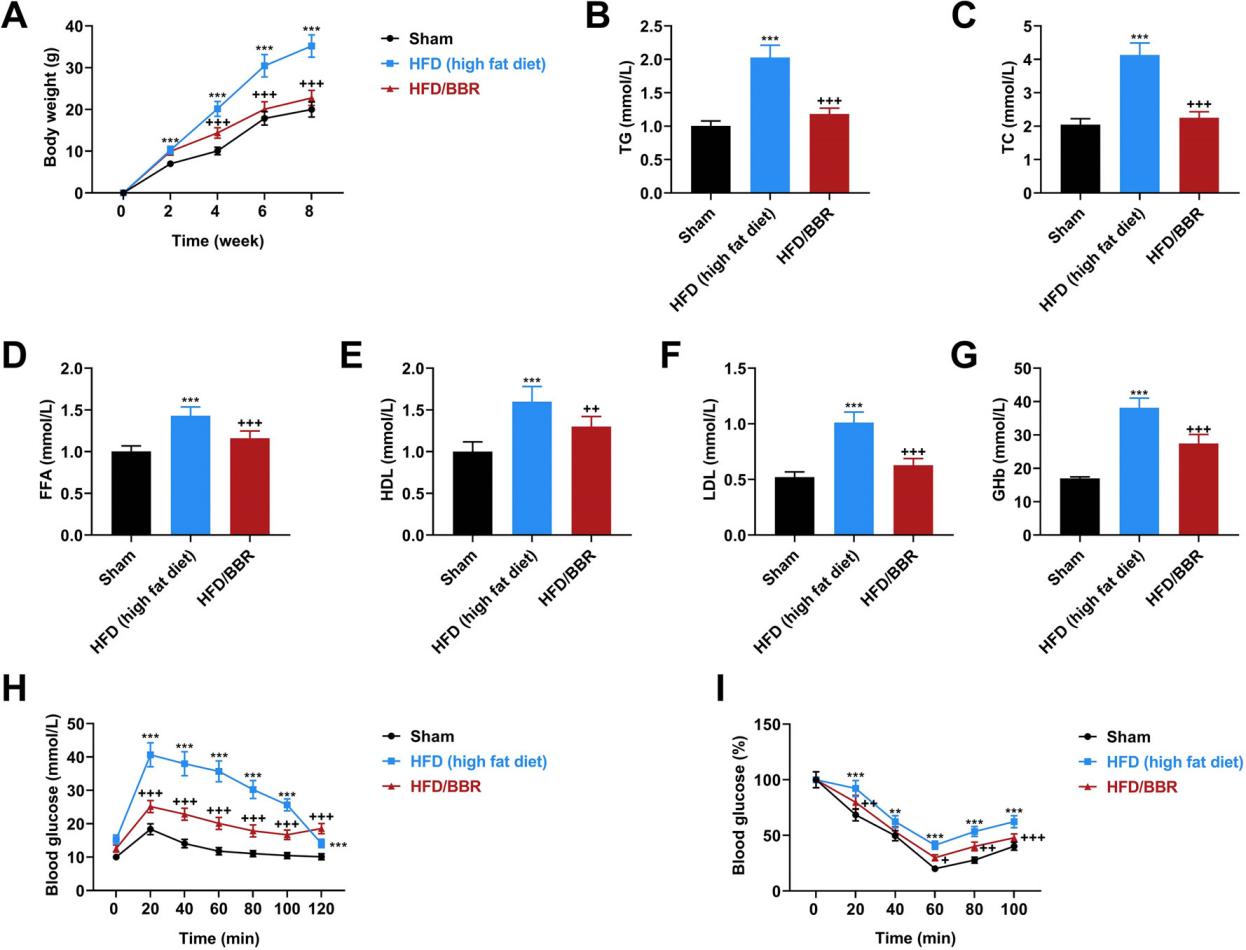
Effects of BBR on weight, biochemical indicators as well as tolerance of glucose and insulin in obese mice. (**A**) Total mouse weight during obesity modeling (high-fat diet) and BBR administration (50 mg/kg), n=18. (**B**-**G**) Biochemical indicators, including TG, TC, FFA, HDL, LDL, and GHb of blood samples from obese mice with/without BBR administration. Blood glucose levels of obese mice with/without BBR administration following glucose injection (**H**) and insulin injection (**I**) in tolerance tests. Values are presented as the means ± SD from at least three independent experiments. **Note: ** **p < 0.01, ***p < 0.001 *vs.* Sham; ^+^*p* < 0.05, ^++^*p* < 0.01, ^+++^*p* < 0.001 *vs.* HFD (high fat diet). **Abbreviations:** BBR, berberine; TG, triglyceride; TC, total cholesterol; FFA, free fatty acid; HDL, high-density lipoprotein; LDL, low-density lipoprotein.

All data in this study were obtained from three independent assays and expressed as mean ± standard deviation by GraphPad Prism 8 (GraphPad, Inc., La Jolla, California, USA). Data among multiple groups were analyzed by two-way analysis of variance (Figs. **1A**, **G**, **H**), and others were analyzed by one-way analysis of variance. The statistical significance was defined when the *p*-value was below the threshold of 0.05.

## 3 RESULTS

### 3.1 BBR Reduced Weight and Improved Glucolipid Metabolism of Obese Mice

To investigate the effects of BBR on glucolipid metabolism, mouse models of obesity were established by HFD induction, during which BBR was administered to mice. As shown in Fig. (**1A**), compared with the normal diet in the Sham group, HFD significantly increased the weight of mice in the HFD group at 2, 4, 6, and 8 weeks (*p* < 0.001). BBR administration markedly blocked the HFD-induced weight increase of mice at 4, 6, and 8 weeks (Fig. (**1A**), (*p* < 0.001). The biochemical indicators, TG, TC, FFA, HDL, LDL, and GHb levels in serum were determined to be higher in HFD group mice with obesity compared to Sham group mice (Figs. **1B**-**G**), (*p* < 0.001), and these biochemical indicators were reversed in BBR-treated mice (Figs. **1B**-**G**), (*p* < 0.01). Results of glucose and insulin tolerance tests showed that compared with sham mice, obese mice had the characteristics of glucose intolerance and insulin resistance (Figs. **1H**, **I**), (*p* < 0.01), while BBR administration improved these conditions of obese mice (Figs. **1H**, **I**), (*p* < 0.05) at 20 min -120 min (glucose) and 20 min - 100 min. The ameliorating effects of BBR administration suggest that glucolipid metabolism may be, in part, regulated by BBR.

### 3.2 BBR and IRE1 Inhibitors Reversed Severe Inflammation and Unbalanced Glucolipid Metabolism in Insulin-Resistant Adipocytes

**Fig. (2):**
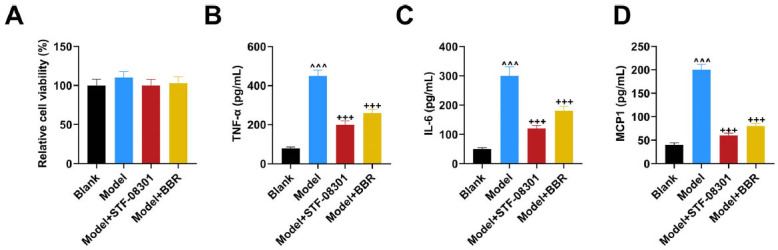
Effects of BBR and IRE1 inhibitors on viability and inflammatory factors in insulin-resistant adipocytes. (**A**) Relative viability of insulin-resistant adipocytes with/without IRE1 inhibitor STF-08301 or BBR treatment (cell counting kit-8 assay). (**B**-**D**) Levels of inflammatory factors in the culture supernatant of insulin-resistant adipocytes with/without IRE1 inhibitor STF-08301 or BBR treatment (ELISA). Values are presented as the means ± SD from at least three independent experiments. **Note: **^^^^^*p* < 0.001 *vs.* Blank; ^+++^*p* < 0.001, *vs.* Model. **Abbreviations:** ELISA, enzyme-linked immunosorbent assay; TNF-α, tumor necrosis factor alpha; IL-6, interleukin-6; MCP1, monocyte chemoattractant protein1; IRE1, inositol requiring enzyme 1.

**Fig. (3):**
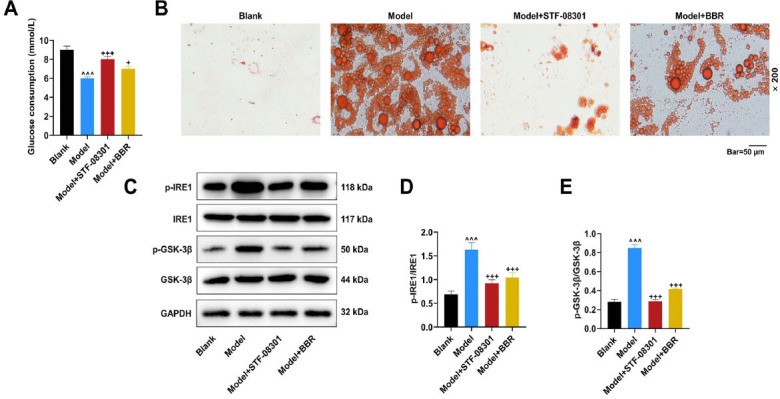
Effects of BBR and IRE1 inhibitors on glucose consumption, lipid droplet generation, and IRE1/GSK-3β axis in insulin-resistant adipocytes. (**A**) Glucose consumption in insulin-resistant adipocytes with/without IRE1 inhibitor STF-08301 or BBR treatment was determined by specific kits (glucose oxidase method). (**B**) Oil red O staining was used to observe lipid droplets in insulin-resistant adipocytes with/without IRE1 inhibitor STF-08301 or BBR treatment at the magnification of ×200 and scale of 50 µm. (**C**-**E**) Relative expression levels of IRE1/GSK-3β axis-associated proteins were tested by Western blot, with GAPDH as the loading control. Values are presented as the means ± SD from at least three independent experiments. **Note: **^^^^^*p* < 0.001 *vs.* Blank; ^+^*p* < 0.05, ^+++^*p* < 0.001 *vs.* Model. **Abbreviations:** GSK-3β, glycogen synthase kinase 3 beta.

To explore the specific mechanism of BBR in glucolipid metabolism, *in vitro*, ELISA experiments were performed in insulin-resistant model adipocytes for TNF-α, IL-6, and MCP1 in cultured 3T3-L1 cells. Based on our hypothesis of BBR targeting the IRE1/GSK-3β axis, an IRE1 inhibitor, STF-08301, was also included in this study. Firstly, we verified that BBR and STF-08301 had no toxic effect on insulin-resistant adipocytes (Fig. **2A**). Afterward, cellular inflammation was assessed *via* measurement of inflammatory factors. It was observed that TNF-α, IL-6, and MCP1 levels in the culture supernatant of insulin-resistant adipocytes were elevated in the Model group, compared with normal cells in the Blank group (Figs. **2B**-**D**), (*p* < 0.001). BBR and STF-08301 treatment inhibited inflammation of insulin-resistant adipocytes *via* diminishing levels of TNF-α, IL-6, and MCP1 (Figs. **2B**-**D**), (*p* < 0.001). In addition, the glucolipid metabolism ability of insulin-resistant adipocytes was tested in glucose assay and oil O staining. Notably, glucose consumption was decreased in the Model group, as compared with that in the Blank group (Fig. **3A**), (*p* < 0.001). In comparison with that in the Model group, glucose consumption was increased at various degrees in Model+STF-08301 and Model+BBR groups (Fig. **3A**), (*p* < 0.05). As shown in Fig. (**3B**), there were vast lipid droplets in insulin-resistant adipocytes of the Model group, while the number of lipid droplets declined in insulin-resistant adipocytes of the Model+STF-08301 and Model+BBR groups.

### 3.3 BBR and IRE1 Inhibitors Blocked IRE1/GSK-3β Axis in Insulin-Resistant Adipocytes

The relative expression levels of proteins in the IRE1/GSK-3β axis were analyzed by western blot analysis to assess the regulation of BBR on the IRE1/GSK-3β axis at the molecular level. p-IRE1/IRE1 and p-GSK-3β/GSK-3β were upregulated in model adipocytes compared with control cells (Figs. **3C**-**E**), (*p* < 0.001), suggesting the activation of the IRE1/GSK-3β axis. Following BBR and STF-08301 treatments, it was observed that p-IRE1/IRE1 and p-GSK-3β/GSK-3β values were downregulated in insulin-resistant adipocytes (Figs. **3C**-**E**), (*p* < 0.001). These findings demonstrated that BBR played a similar role as an IRE1 inhibitor in inhibiting the IRE1/GSK-3β axis in insulin-resistant adipocytes.

## 4 DISCUSSION

Insulin resistance is an initial event of unbalanced glucolipid metabolism in the context of obesity [8, 9]. In this study, it was discovered that BBR reduced weight and ameliorated glucolipid metabolism of HFD-induced obese mice, as reflected by the improvement of biochemical indicators as well as glucose intolerance and insulin resistance. ERS and inflammation of adipocytes are involved in insulin resistance in the occurrence and development of obesity [6, 7, 10-12]. Here, it was verified that BBR repressed the secretion of inflammatory factors, promoted glucolipid metabolism, and blocked the IRE1/GSK-3β axis in insulin-resistant adipocytes, with the same role as the IRE1 inhibitor. These findings suggested the mechanism by which BBR ameliorated insulin resistance *via* inhibiting IRE1/GSK-3β axis.

HFD is a common method of obesity induction for experimental animals and is widely used in research on diabetes mellitus and metabolic syndrome [28]. A previous study has reported that HFD elevates mouse weight and upregulates blood TG, TC, FFA, LDL, and HDL cholesterol [29]. Similarly, we observed that HFD induced an increase in the weight of the mouse and elevation in the serum TG, TC, FFA, LDL, and HDL and strengthened glucose consumption in glucose and insulin tolerance tests. Baldassano *et al.* also mentioned weakened glucose tolerance and exogenous insulin sensitivity as well as plasma lipid metabolic profile in HFD-induced obese mice [30]. More importantly, in this study, BBR administration during HFD feeding alleviated changes in these indicators of glucolipid metabolism. In fact, the regulatory role of BBR in glucolipid metabolism has been confirmed in various publications. In the study on BBR in type 2 diabetes mellitus, BBR has been demonstrated to decrease blood glucose and mitigate the impaired glucose tolerance of Zucker diabetic fatty rats [31]. Moreover, the report by Fang *et al.* indicated that BBR alleviates fasting blood glucose and glucose tolerance and decreases weight, TG, and TC of mice with disturbances in glucose and lipid metabolism [32], which is in line with our study. Our study suggests that BBR had the ability to reverse insulin tolerance to regulate glucolipid metabolism in obese mice.

Chronic adipose tissue inflammation is a key component of obesity-induced insulin resistance and type 2 diabetes [33]. Also, IRE1/GSK-3β axis-mediated ERS is likely to be related to metabolic inflammation in adipocytes, as described above. Hence, we constructed insulin-resistant adipocytes *via* Dex stimulation to further explore whether BBR displayed a role in repressing inflammation *via* the IRE1/GSK-3β axis. Eventually, we found that levels of TNF-α, IL-6, and MCP1 in culture supernatant and the number of lipid droplets were increased, and glucose consumption was decreased in insulin-resistant adipocytes, suggesting the relationship between inflammation and aberrant glucolipid metabolism induced by insulin resistance. *In vivo* studies by others have identified that obesity, adipose tissue inflammation, and insulin resistance appear simultaneously in mice with HFD [34, 35]. Of note, BBR and IRE1 inhibitors downregulated secretion of inflammatory factors and lipid droplet generation, as well as elevated glucose consumption of insulin-resistant adipocytes, indicating the palliative roles of BBR and IRE1 inhibitors in inflammation and insulin resistance of adipocytes. In fact, a clinical study has reported that inflammation in adipose tissue is implicated in systemic inflammation and insulin resistance [36]. Accordingly, we have reason to believe that BBR has an anti-obesity role due to the reversal of insulin resistance of adipocytes.

IRE1 has been reported to link ERS to obesity-associated inflammation and insulin resistance in various research works [14, 37, 38]. As mentioned, in obesity and diabetes mellitus, anoxic 3T3-L1 adipocytes activate IRE1-mediated UPR to repress the expression of adiponectin, act as an anti-inflammatory and anti-apoptotic agent, and improve insulin sensitivity [39]. It has been discovered that ERS-induced IRE1 activation regulates proinflammatory cytokine gene expression *via* GSK-3β activation [16]. Besides, it has been reported that increased p-IRE1/IRE1 and p-GSK-3β/GSK-3β levels were closely related to insulin resistance, and the inhibition of insulin resistance can be induced by regulating IRE1/GSK-3β axis [40, 41]. Consistently, we observed the increase in p-IRE1/IRE1 and p-GSK-3β/GSK-3β in insulin-resistant adipocytes, which were similar to the findings of previous studies. In addition, the upregulated p-IRE1/IRE1 and p-GSK-3β/GSK-3β levels were negated by BBR and IRE1 inhibitors, which implied that BBR functioned as an IRE1 inhibitor to suppress IRE1/GSK-3β axis, thereby inhibiting the insulin resistance of adipocytes.

## CONCLUSION

This study contributes to a better understanding of the mechanism of BBR regulating glucolipid metabolism. Specifically, BBR initiates an anti-inflammation role by blocking the IRE1/GSK-3β axis. This discovery hints at the great potential of BBR in reversing insulin resistance in obesity. In addition, PKC has been proven to be closely related to IRE1/GSK-3β axis-mediated ERS [42]. Nevertheless, our study has not yet detected the dose-dependent effects of BBR and other protein expressions related to the IRE1/GSK-3β signaling pathway (*e.g.,* PKC), which are the shortcomings of this study and will be supplemented in the future.

## Data Availability

The analyzed data sets generated during the study are available from the corresponding author upon reasonable request.

## LIST OF ABBREVIATIONS

ERS: Endoplasmic Reticulum Stress
HFD: High-Fat Diet
IL-1: Interleukin-1
IRE1: Inositol Requiring Enzyme 1
MCP1: Monocyte Chemoattractant Protein1
TNF-α: Tumor Necrosis Factor-Alpha
UPR: Unfolded Protein Response
